# Conditional Genetic Interactions of *RTT107*, *SLX4*, and *HRQ1* Reveal Dynamic Networks upon DNA Damage in *S. cerevisiae*

**DOI:** 10.1534/g3.114.011205

**Published:** 2014-04-02

**Authors:** Grace P. Leung, Maria J. Aristizabal, Nevan J. Krogan, Michael S. Kobor

**Affiliations:** * Centre for Molecular Medicine and Therapeutics, Child and Family Research Institute, Department of Medical Genetics, University of British Columbia, Vancouver, B.C. Canada V5Z 4H4; † Department of Cellular and Molecular Pharmacology, California Institute for Quantitative Biomedical Research, University of California, San Francisco, San Francisco, California 94158

**Keywords:** genetic interaction profiles, DNA damage response, helicase, conditional interactions

## Abstract

The DNA damage response (DDR) is a dynamic process that is crucial for protecting the cell from challenges to genome integrity. Although many genome-wide studies in *Saccharomyces cerevisiae* have identified genes that contribute to resistance to DNA-damaging agents, more work is needed to elucidate the changes in genetic interaction networks in response to DNA lesions. Here we used conditional epistatic miniarray profiling to analyze the genetic interaction networks of the DDR genes *RTT107*, *SLX4*, and *HRQ1* under three DNA-damaging conditions: camptothecin, hydroxyurea, and methyl methanesulfonate. Rtt107 and its interaction partner Slx4 are targets of the checkpoint kinase Mec1, which is central to the DDR-signaling cascades. Hrq1 recently was identified as a novel member of the RecQ helicase family in *S. cerevisiae* but is still poorly characterized. The conditional genetic networks that we generated revealed functional insights into all three genes and showed that there were varied responses to different DNA damaging agents. We observed that *RTT107* had more genetic interactions under camptothecin conditions than *SLX4* or *HRQ1*, suggesting that Rtt107 has an important role in response to this type of DNA lesion. Although *RTT107* and *SLX4* function together, they also had many distinct genetic interactions. In particular, *RTT107* and *SLX4* showed contrasting genetic interactions for a few genes, which we validated with independently constructed strains. Interestingly, *HRQ1* had a genetic interaction profile that correlated with that of *SLX4* and both were enriched for very similar gene ontology terms, suggesting that they function together in the DDR.

Mapping of genetic interactions has been a valuable and powerful approach to reveal connections within complex biological systems (Baryshnikova *et al.* 2013). Much of this work has been done in *Saccharomyces cerevisiae* because of the tools in place to create double mutants and the availability of vast arrays of mutant libraries. Although providing great biological insights, most screens to date have been conducted under unperturbed growth conditions, whereas many networks in cells respond to environmental stimuli.

A significant type of environmental stimuli is DNA damage, which can be caused by external factors, such as exposure to genotoxins or ultraviolet light, or internal factors, such as replication fork stalling or DNA polymerase error (Lindahl 1993). Cells are constantly exposed to these insults that, if not properly repaired, may compromise genomic integrity or ultimately lead to cell death. Because of the vital importance of genomic integrity, cells have complex mechanisms to regulate the DNA damage response. DNA damage is detected by sensors, which trigger a signaling cascade, leading to the activation of the kinases Mec1 and Tel1, the yeast homologs of mammalian ATR (ATM and Rad 3-related) and ATM (ataxia-telangiectasia mutated). These kinases in turn elicit various cellular responses, including cell-cycle arrest, DNA repair, apoptosis, and/or DNA damage-induced transcriptional program (Putnam *et al.* 2009; Finn *et al.* 2012).

One of the downstream phosphorylation targets of Mec1 is Rtt107/Esc4, which is required for reinitiating replication after repair of alkylating DNA damage (Rouse 2004; Roberts *et al.* 2006). Deletion of the *RTT107* gene results in hypersensitivity to DNA-damaging agents such as the DNA-alkylating agent methyl methane-sulfonate (MMS), the nucleotide reductase inhibitor hydroxyurea (HU), and the topoisomerase I poison camptothecin (CPT) (Chang *et al.* 2002; Rouse 2004; Parsons *et al.* 2006; Roberts *et al.* 2006). Rtt107 contains several BRCT (BRCA1 C-terminal) homology domains, which often serve as phosphobinding modules to recruit signaling complexes and repair factors to DNA damage-induced lesions (Rouse 2004; Mohammad and Yaffe 2009). Consistent with a role as a scaffold for protein-protein interactions during the DNA damage response, Rtt107 interacts with a number of DNA repair and recombination proteins and is recruited to sites of DNA lesions (Chin *et al.* 2006; Roberts *et al.* 2006, 2008; Ohouo *et al.* 2010; Leung *et al.* 2011; Ullal *et al.* 2011).

The best-characterized Rtt107-interacting partner is the replication-specific endonuclease Slx4, which interacts with the N-terminal BRCT domains of Rtt107 (Roberts *et al.* 2006). Slx4 is required for Mec1-dependent phosphorylation of Rtt107 and, like Rtt107, facilitates resumption of DNA replication after DNA damage (Roberts *et al.* 2006). However, it has become clear over the last few years that Rtt107 also has Slx4-independent functions, and vice versa. Consistent with this, the defects in DNA damage response are generally more severe in *rtt107*∆ mutants than in *slx4*∆ mutants, and *rtt107*∆ *slx4*∆ double mutants are more sensitive to MMS than either of the single mutants (Roberts *et al.* 2006).

Although Slx4 has been studied in the context of its interaction with Rtt107, the *SLX4* gene was first identified in a synthetic lethal screen with *SGS1*, which encodes for a RecQ helicase (Mullen *et al.* 2001). DNA helicases represent an important class of enzymes involved in the DNA damage response and have roles in recognition of DNA damage, DNA recombination, and stabilization of stalled replication forks (Brosh 2013). In *S. cerevisiae*, Sgs1 was thought to be the only RecQ helicase family member until recently, when Hrq1 was identified as a novel RecQ helicase (Barea *et al.* 2008; Kwon *et al.* 2012). The functions of Hrq1 have only been preliminarily characterized, but based on the relationship between Slx4 and Sgs1, Hrq1 may also have linkages to Slx4 that have yet to be uncovered.

Initial genome-wide studies to characterize genetic function in response to DNA damage measured the fitness of deletion mutants exposed to a variety of genotoxic insults (Giaever *et al.* 2004; Parsons *et al.* 2006; Hillenmeyer *et al.* 2008). However, these studies only evaluated the requirement of single genes for resistance to DNA-damaging agents, whereas the effects on genetic networks were only studied in a small directed screen (St Onge *et al.* 2007). Two recent studies used genetic interaction mapping to gain new insights into the DNA damage response (Bandyopadhyay *et al.* 2010; Guenole *et al.* 2013). In the initial study, all possible double mutants of 418 genes were created and exposed to MMS to evaluate changes in the genetic interaction network (Bandyopadhyay *et al.* 2010). Using this approach, the authors demonstrated that differential genetic interactions are better able to reveal functions in the DNA damage response and identified new roles for several genes. A follow-up study expanded on this work and interrogated 55 query genes crossed to a library of more than 2000 genes in MMS, CPT, and zeocin conditions (Guenole *et al.* 2013). Analysis of the differential genetic networks revealed several genes that were hubs of genetic interactions, and additional experiments demonstrated that these genes had novel roles in the DNA damage response.

Here we use a similar approach of measuring conditional genetic interactions to study further the functions of Rtt107, Slx4, and Hrq1. We analyzed the significantly interacting gene pairs to identify those that emerged or changed in response to DNA damage. Overall, *RTT107* exhibited more genetic interactions than *SLX4* or *HRQ1* in CPT conditions, indicating an important role for Rtt107 in responding to CPT. Furthermore, *SLX4* and *RTT107* showed distinct, and sometimes even opposing, genetic interactions, even though the protein products exist at least in part as a complex in the cell. Interestingly, the interaction profile and enriched gene ontology terms for *HRQ1* most closely resembled that of *SLX4*, suggesting that they have overlapping functions in the DNA damage response.

## Material and Methods

### Yeast strains

All yeast strains used in this study are listed in Supporting Information, Table S1 and created using standard yeast genetic techniques (Ausubel 1987). Complete gene deletions were achieved using one-step gene integration of polymerase chain reaction-amplified modules (Longtine *et al.* 1998). Mutants for conditional epistatic miniarray profiling (cE-MAP) screens were constructed in the BY4742 background, whereas all other strains were constructed in the W303-1A background.

### cE-MAP

**Figure 1:**
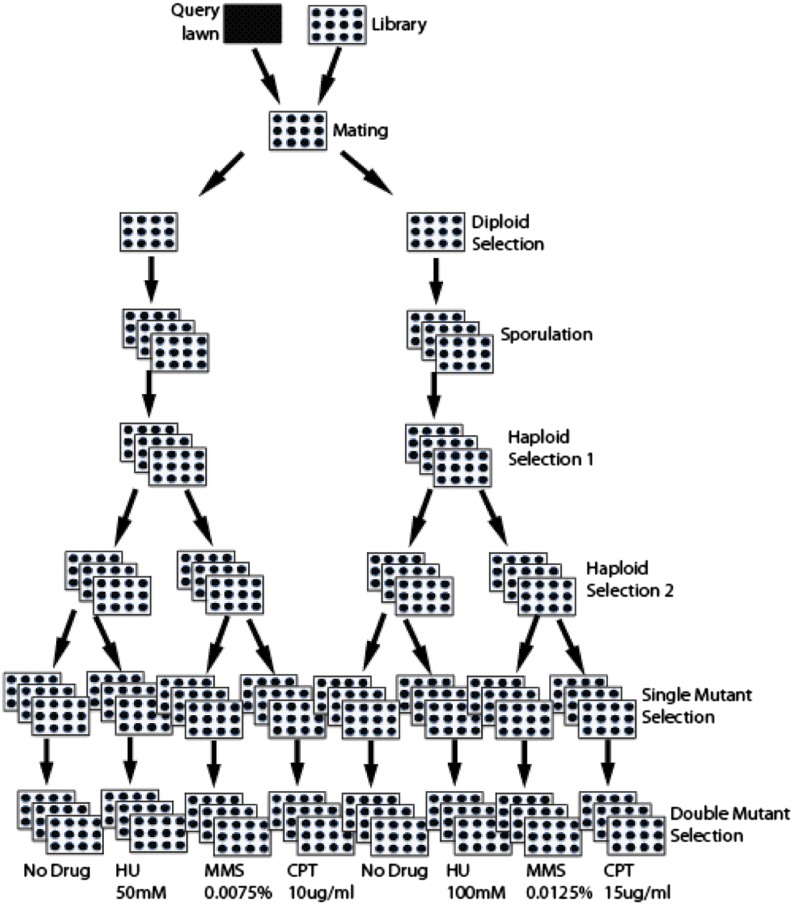
Schematic diagram of the conditional epistatic miniarray profiling (cE-MAP) workflow. The number of plates was expanded at various points in the process to accommodate for the multiple conditions tested. All screens were performed three times. HU, hydroxyurea; MMS, methyl methane-sulfonate; CPT, camptothecin.

cE-MAP screens were performed and normalized as described previously (Collins *et al.* 2010), with the exception that we expanded the number of plates per query strain to accommodate all drug conditions tested (see Figure 1). In brief, deletion mutants of the query genes were crossed, using a Singer robot, to a library of 1536 mutants covering a number of categories, including kinases/phosphatases and chromatin biology. We used 10 and 15 μg/mL of CPT, 50 and 100 mM of HU, and 0.0075 and 0.0125% of MMS (Sigma-Aldrich), along with two no-drug controls. All strains and conditions were screened in triplicate. Complete cE-MAP profiles can be found in File S1.

Differential S-scores were calculated by subtracting the S-score in yeast extract peptone dextrose from the S-score in each drug condition for each gene pair, and converted to Z-scores. Corresponding *p*-values were corrected for multiple testing using the fdrtool R package (Strimmer. 2008) and conditional genetic interactions were considered significant for *q*-values < 0.05. cE-MAP networks were visualized with Cytoscape (Cline *et al.* 2007).

### Growth and DNA damage sensitivity assays

Overnight cultures grown in yeast extract peptone dextrose were diluted to 0.5 OD600. The cells were tenfold serially diluted and spotted onto solid yeast extract peptone dextrose plates or plates with MMS, CPT, or HU (Sigma-Aldrich) at the indicated concentrations. The plates were then incubated at 30° for 2 d and subsequently photographed.

### Gene Ontology (GO) analysis

The Database for Annotation, Visualization, and Integrated Discovery was used for GO term enrichment analysis (Dennis *et al.* 2003). For each query gene, a list of significantly interacting genes was compiled that included all drug conditions and both positively and negatively interacting genes. Multiple testing correction was done using the Benjamini method, and enriched GO terms were considered significant for *q*-values < 0.05.

## Results

### Genetic interaction profiles were considerably altered when exposed to DNA-damaging agents

To gain a global understanding of the functions of Rtt107, Slx4, and Hrq1, we measured genetic interactions with representative genes across the genome under three DNA damaging conditions (MMS, HU, and CPT). To achieve this, we used a version of the synthetic genetic array technology, the epistatic miniarray profile (E-MAP), to map genetic interactions for *RTT107*, *SLX4*, and *HRQ1* under different DNA-damaging conditions, which we termed cE-MAP (Tong *et al.* 2001; Baryshnikova *et al.* 2010; Collins *et al.* 2010). During the mutant selection process, the plates were expanded to accommodate the four different conditions tested and to measure each condition with three technical replicates (see Figure 1 for workflow).

**Figure 2:**
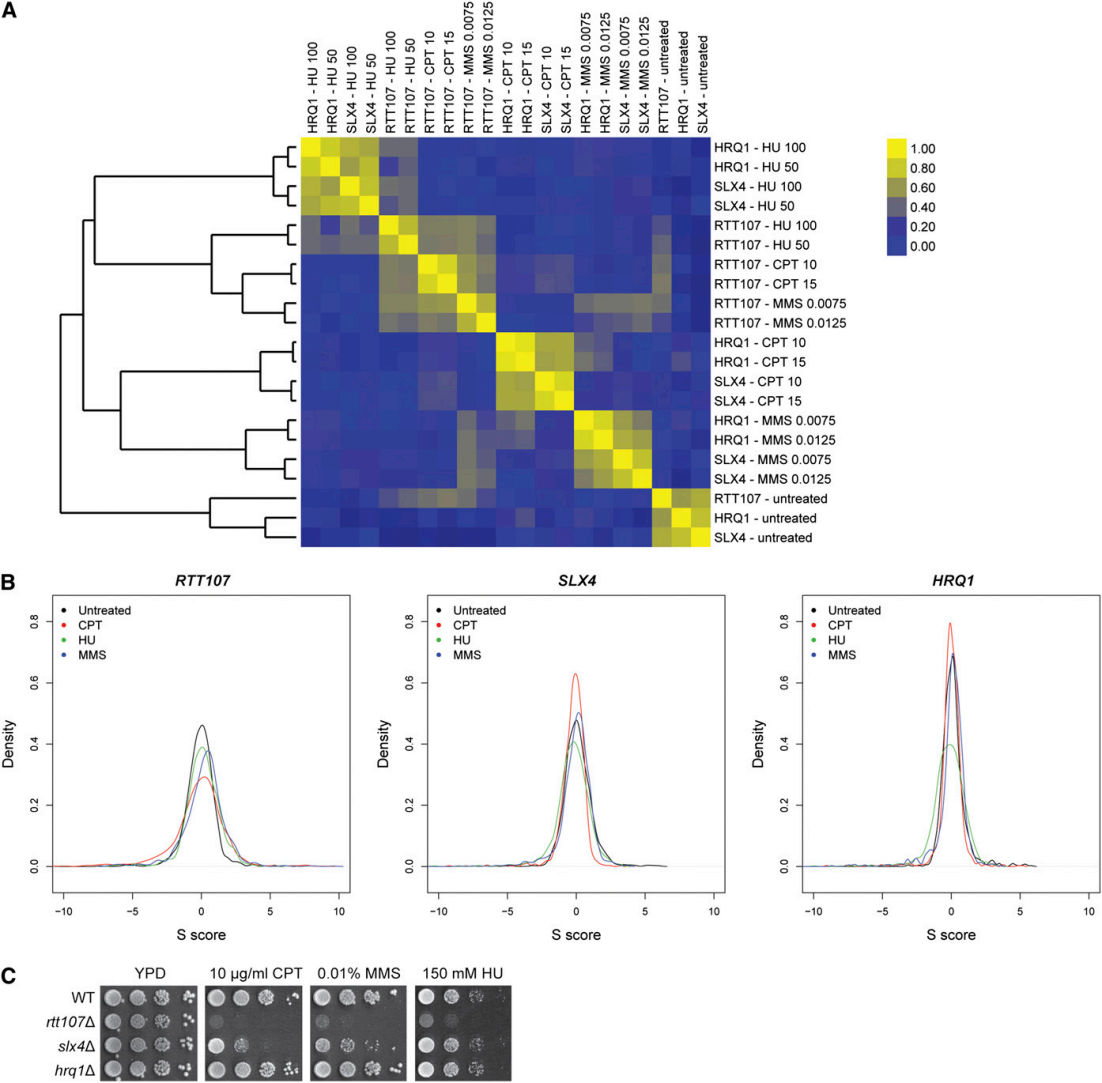
Genetic interaction profiles changed in response to DNA-damaging agents. (A) Pearson’s correlation revealed that the genetic interaction profiles of *RTT107* were more similar to one another regardless of the drug condition, whereas *SLX4* and *HRQ1* were more similar to one another. (B) Density plots of the S-scores for each query gene showed a broader distribution of S-scores for *RTT107* than *SLX4* or *HRQ1*. (C) *rtt107*∆ mutants were more sensitive to DNA-damaging agents than *slx4*∆ or *hrq1*∆. 10-fold serial dilutions of the indicated strains were plated onto media containing the indicated drugs.

The analysis pipeline for E-MAP calculates S-scores, which reflect both the strength of the genetic interaction and the statistical confidence (Collins *et al.* 2010). For a broad assessment of how DNA damage affected the genetic networks, we first calculated the Pearson’s correlations of the query genes’ S-score profiles under the three drug conditions (Figure 2A). Supporting the idea that genetic networks respond significantly to external stimuli, the genetic interaction profiles generated under unperturbed growth conditions clustered away from the profiles generated under DNA-damaging conditions. Strikingly, the profiles for *RTT107* in DNA-damaging conditions were closely correlated, regardless of the type of DNA insult. In contrast, the profiles for *SLX4* and *HRQ1* clustered together by the type of DNA-damaging agent. In addition to these major patterns, the genetic interaction profiles of all query genes under the same type of DNA-damaging agent also were positively correlated to one another (for example, see the positive correlations between the *RTT107*, *SLX4*, and *HRQ1* profiles in MMS). Finally, we observed that the two concentrations of drug used produced very similar profiles; thus, we averaged the scores of the two concentrations. This improved the confidence of our S-scores since they were represented by six replicates instead of three; these sets of average S-scores were used for all further analyses.

According to previously published thresholds, S-scores lower than -2.4 or greater than 2.0 are considered significant genetic interactions (Collins *et al.* 2010). Consistent with the dynamic nature of the DNA damage response, the distribution of the S-scores for each query gene changed significantly between the unperturbed growth conditions and the DNA-damaging conditions (Figure 2B and Figure S1). For example, under HU conditions, the S-score distribution for each query gene became broader, indicating that the number and strength of the genetic interactions increased (Kolmogorov-Smirnov test, two-sided, *p*-value < 1.5 × 10^−7^ for all comparisons). Strikingly, *rtt107*∆ mutants had the broadest distribution of S-scores of the three query mutants, *i.e.*, the most significant genetic interactions, followed by *slx4*∆ mutants, then *hrq1*Δ mutants (Figure 2B). We note that this was consistent with the relative DNA damage sensitivity of these mutants (Figure 2C). The distribution of the S-scores of all conditions for all three query genes were significantly different from one another, although the distributions for *SLX4* and *HRQ1* looked more similar to one another than to that of *RTT107* (Kolmogorov-Smirnov test, two-sided, *p*-value < 7.8 × 10^−16^ for all comparisons, Figure S1D).

### *RTT107* exhibited more conditional genetic interactions than *SLX4* or *HRQ1*

**Figure 3:**
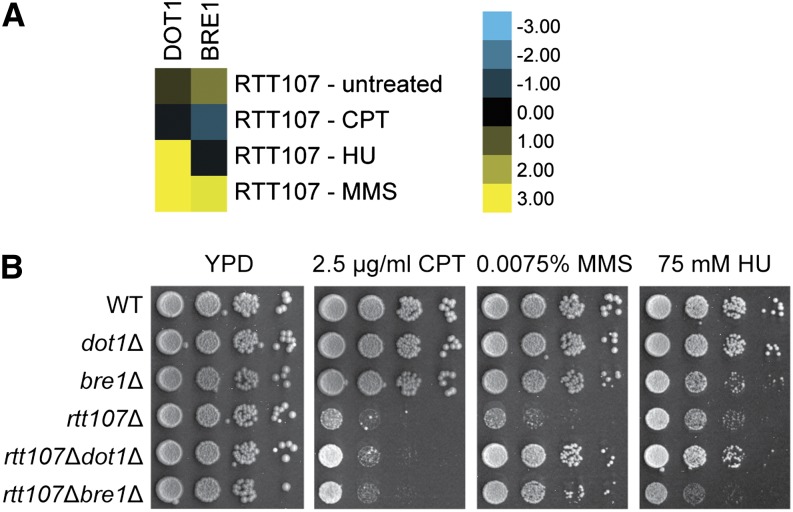
cE-MAP data recapitulated the drug-specific genetic interactions between *RTT107* and *DOT1* or *BRE1*. (A) Shown are subsets of cE-MAP data. Blue and yellow represent negative and positive genetic interactions, respectively. (B) 10-fold serial dilutions of the indicated strains were plated onto media containing the indicated drugs.

To identify genetic interactions under DNA damage conditions that were significantly different from unperturbed growth conditions, we adapted a published method (Bandyopadhyay *et al.* 2010). Specifically, the differences between the S-score in the DNA damage condition and the S-score in unperturbed growth conditions for each gene pair were subtracted from the average of all the differential scores and divided by the standard error to calculate a Z score. Genes with significant Z scores after correcting for multiple testing are listed in Table 1 (*q*-value < 0.05). In total there were 569 gene pairs found to have significant differential interaction in the drug conditions tested. Of these, 378 were negative interactions (DNA damage-induced sickness or lethality), and 191 were positive interactions (DNA damage-induced epistasis or suppression). To test the reliability of this approach, we looked for known condition-specific genetic interactions. Consistent with our previously published results, deletion of *DOT1* and *BRE1* suppressed the DNA damage sensitivity of *rtt107*∆ in MMS (Levesque *et al.* 2010). Furthermore, the suppression by *dot1*∆ was additionally observed in HU but not in CPT, whereas the suppression by *bre1*∆ was limited to MMS, in both the cE-MAP data and the independently constructed mutants (Figure 3).

**Table 1:** Genes that showed condition-specific interactions with query genes that were statistically significant after multiple test correction (*q* < 0.05)

Query	Drug	Interaction	Significant Genes
*HRQ1*	CPT	Negative	*ASF1*, *CHL1*, *CLB5*, *CTF4*, *DCC1*, *DDC1*, *MMS1*, *MMS22*, *MRE11*, *PBY1*, *RAD17*, *RAD24*, *RAD52*, *RAD54*, *RAD55*, *RAD57*, *RAD59*, *RTT101*, *RTT109*, *SAE2*
*HRQ1*	CPT	Positive	*CYC8*, *GMH1*, *PDA1*
*HRQ1*	HU	Negative	*ARP4*, *ASF1*, *BAS1*, *BMH1*, *BRE1*, *CLB5*, *ERG5*, *ERJ5*, *GCN1*, *GCN20*, *GET2*, *GNP1*, *HPC2*, *IRA2*, *LAT1*, *LGE1*, *LST4*, *MET18*, *MFT1*, *MKS1*, *MRC1*, *MRE11*, *NPR1*, *PBS2*, *PDB1*, *PDE2*, *PMR1*, *POL32*, *RAD52*, *RAD54*, *RAD55*, *RAD57*, *RIM21*, *RPL34B*, *RPS21B*, *RTF1*, *RTG3*, *RTT109*, *SDC1*, *SEC22*, *SEC66*, *SEC72*, *SGF73*, *SNX4*, *SPF1*, *SUA7*, *SWD1*, *SWD3*, *SWI4*, *SYC1*, *UBA3*, *UBP15*, *UBP6*, *URE2*, *VPS8*, *YTA7*
*HRQ1*	HU	Positive	*AIM21*, *APS3*, *ARO1*, *ARP7*, *BTS1*, *BUL1*, *CAP1*, *COQ2*, *CUE3*, *CYC8*, *CYT1*, *DCR2*, *FEN1*, *HOS2*, *MSS18*, *NGL2*, *PET130*, *THI6*, *YMR102C*
*HRQ1*	MMS	Negative	*DAL81*, *FKS1*, *GFD1*, *GFD2*, *GNP1*, *MMS2*, *MOG1*, *MPH1*, *MRE11*, *MSH4*, *PBY1*, *PET18*, *PHO5*, *POL32*, *RAD10*, *RAD18*, *RAD27*, *RAD59*, *REV1*, *REV3*, *REV7*, *RVS161*, *SAK1*, *SCS7*, *SRO9*, *STP1*, *TRS33*, *UBC13*, *VMA21*, *YGL081W*, *YSY6*
*HRQ1*	MMS	Positive	*CLB5*, *HST3*, *ILM1*, *KAP122*, *PMR1*, *SUR4*
*RTT107*	CPT	Negative	*AGE2*, *BCK1*, *BFA1*, *BUB2*, *CHS5*, *CLB2*, *CLB5*, *COG5*, *CRN1*, *CSG2*, *DCC1*, *DDC1*, *DEP1*, *ECM33*, *ELA1*, *ERV14*, *FET3*, *FKS1*, *FUN30*, *GAS1*, *HPC2*, *IRA2*, *IRC21*, *KEX2*, *LAS21*, *LEM3*, *LGE1*, *LTE1*, *MAK31*, *NCL1*, *OPI3*, *OST3*, *PAC1*, *PBY1*, *PEP8*, *PFA4*, *PMT1*, *PMT2*, *PPH21*, *PPH3*, *PPM1*, *PRE9*, *PSP2*, *RAD17*, *RAD24*, *RAD54*, *RAD55*, *RAD57*, *RAD61*, *RDH54*, *RDI1*, *REV7*, *RGA1*, *RRD2*, *RTF1*, *RTS1*, *RXT2*, *SAP30*, *SCS7*, *SEC22*, *SEC28*, *SLA1*, *SMI1*, *SMY1*, *SPF1*, *SRO9*, *STV1*, *SUR4*, *SWI4*, *TPK3*, *UBP14*, *VPS24*, *VPS27*, *VPS29*, *VPS35*, *VPS5*, *VPS8*, *VPS9*, *YDR061W*, *YJR088C*, *YLR426W*, *YPL150W*, *ZDS1*
*RTT107*	CPT	Positive	*APQ12*, *ARP7*, *BUB1*, *BUB3*, *CDC28*, *CYC8*, *CYT1*, *DBF2*, *DPB4*, *ELC1*, *ELG1*, *ESS1*, *GAC1*, *GAL80*, *HDA1*, *IKI3*, *IPT1*, *JHD2*, *LIA1*, *MIP1*, *MKK2*, *MMS4*, *MRC1*, *MRT4*, *NAP1*, *NCS2*, *NFI1*, *NOP12*, *OAF1*, *PAP2*, *PEF1*, *POL32*, *PSY4*, *RAD27*, *RPL11B*, *RPL8B*, *RPS1B*, *RPS4A*, *RSC4*, *SAC3*, *SAM37*, *SCT1*, *SFL1*, *SGF29*, *SLC1*, *SMT3*, *SPT2*, *SSN2*, *TED1*, *TOP1*, *TRM10*, *TUB3*, *TUP1*, *UBX4*, *UFO1*, *YCR050C*, *YLR287C*, *YNR004W*
*RTT107*	HU	Negative	*AGE2*, *BAS1*, *BUB2*, *CRN1*, *DUG2*, *GCN1*, *IRA2*, *MAD2*, *NPR1*, *OXR1*, *PBS2*, *PSP2*, *SEC22*, *SER2*, *SLX9*, *SLY41*, *SPF1*, *SUR4*, *SWI4*, *URE2*, *YDL089W*
*RTT107*	HU	Positive	*AEP2*, *AIM26*, *ARO1*, *ARP7*, *BTS1*, *BUB3*, *BUD21*, *BUD6*, *BUL1*, *CDC48*, *CYC8*, *CYK3*, *CYT1*, *DBP1*, *ECM5*, *ERG6*, *HDA1*, *JNM1*, *LAG1*, *MET22*, *NAP1*, *NUP2*, *PEF1*, *PET130*, *PEX17*, *PMR1*, *POL30*, *PPM1*, *RIM101*, *RPN13*, *RPS1B*, *RPS28B*, *RPS30A*, *SCT1*, *SEF1*, *SPE3*, *SPT21*, *SSE1*, *THI6*, *TSA1*, *UBX4*, *UFO1*
*RTT107*	MMS	Negative	*BCK1*, *BFA1*, *CLB2*, *CRN1*, *DDC1*, *DUG2*, *FKS1*, *GFD1*, *GNP1*, *HRT1*, *IRC21*, *LSM7*, *LTE1*, *MBP1*, *MMS2*, *PAC1*, *POL30*, *PPM1*, *PTC2*, *RAD17*, *RAD18*, *RAD27*, *REV7*, *RTS1*, *SCS7*, *SLX9*, *SRO9*, *SRS2*, *STP1*, *TEL1*, *TSA1*, *UBC13*, *VMA21*, *YGL081W*, *YPL041C*
*RTT107*	MMS	Positive	*AIM29*, *APQ12*, *ARO1*, *BMH1*, *BTS1*, *CSM3*, *CYT1*, *DBF2*, *DOT1*, *ELC1*, *FEN1*, *GAL80*, *GSF2*, *HST3*, *ILM1*, *LGE1*, *MRC1*, *MSS18*, *NAM7*, *NPP1*, *PER1*, *RAD52*, *RMD11*, *ROT2*, *RPL8B*, *RPS1B*, *RPS4A*, *RSC4*, *SPT2*, *TEP1*, *TMA23*, *TOP1*, *TUB3*, *UFO1*
*SLX4*	CPT	Negative	*ARP4*, *ASF1*, *CHL1*, *CIK1*, *CLB5*, *CSM3*, *CTF4*, *DCC1*, *DDC1*, *DIA2*, *GAS1*, *LAS21*, *LEM3*, *LGE1*, *MMS1*, *MMS22*, *PBY1*, *PMR1*, *PPH3*, *RAD17*, *RAD24*, *RAD52*, *RAD54*, *RAD55*, *RAD57*, *RAD59*, *RDI1*, *RTT101*, *RTT109*, *SAE2*, *SGF73*, *SRS2*, *STV1*
*SLX4*	HU	Negative	*AGE2*, *AIM32*, *ARL3*, *ARP4*, *BAS1*, *BMH2*, *BRE1*, *CLB5*, *COG5*, *CWH41*, *DDC1*, *ECM30*, *ERG5*, *ERJ5*, *FKH2*, *GCN1*, *GCN20*, *GEF1*, *GNP1*, *HCM1*, *HPC2*, *INP53*, *IRA2*, *LGE1*, *LST4*, *MDS3*, *MRE11*, *NPR1*, *OXR1*, *PBS2*, *PBY1*, *PDB1*, *PDE2*, *PFA4*, *PMR1*, *PRE9*, *PSP2*, *QCR10*, *QNQ1*, *RAD54*, *RAD55*, *RAD57*, *RPL41B*, *RPS11A*, *RTF1*, *SDC1*, *SEC22*, *SEC66*, *SGF73*, *SKY1*, *SNG1*, *SPF1*, *STV1*, *SUA7*, *SUR4*, *SWD1*, *SWD3*, *SWI4*, *SYC1*, *SYF2*, *UBP15*, *URE2*, *VPS27*, *YDR061W*, *YER064C*, *YPR063C*, *YSY6*
*SLX4*	HU	Positive	*APS3*, *BTS1*, *BUL1*, *CAP1*, *CDC36*, *COQ2*, *CYT1*, *FEN1*, *HOS2*, *IMP2*, *MDM38*, *MFT1*, *MSS18*, *NGL2*, *PET123*, *PET130*, *RPL43A*, *RPN13*, *SCD6*, *SPT21*, *TAF9*, *TOP1*, *YDL176W*, *YIP3*
*SLX4*	MMS	Negative	*CKB1*, *DIA2*, *ENT4*, *ERV25*, *GFD1*, *GNP1*, *HST3*, *IMP2*, *MMS2*, *MPH1*, *PAC1*, *PET18*, *POL32*, *PPH3*, *PSY3*, *RAD18*, *RAD26*, *RAD27*, *RAD59*, *REV3*, *REV7*, *RRD1*, *SAE2*, *SAP185*, *SCS7*, *STP1*, *TOM7*, *TSR3*, *UBC11*, *UBC13*, *VMA21*, *YGL081W*
*SLX4*	MMS	Positive	*BUD14*, *DOT1*, *MSH4*, *NAM7*, *RPS21B*

CPT, camptothecin; HU, hydroxyurea; MMS, methyl methane-sulfonate.

**Figure 4:**
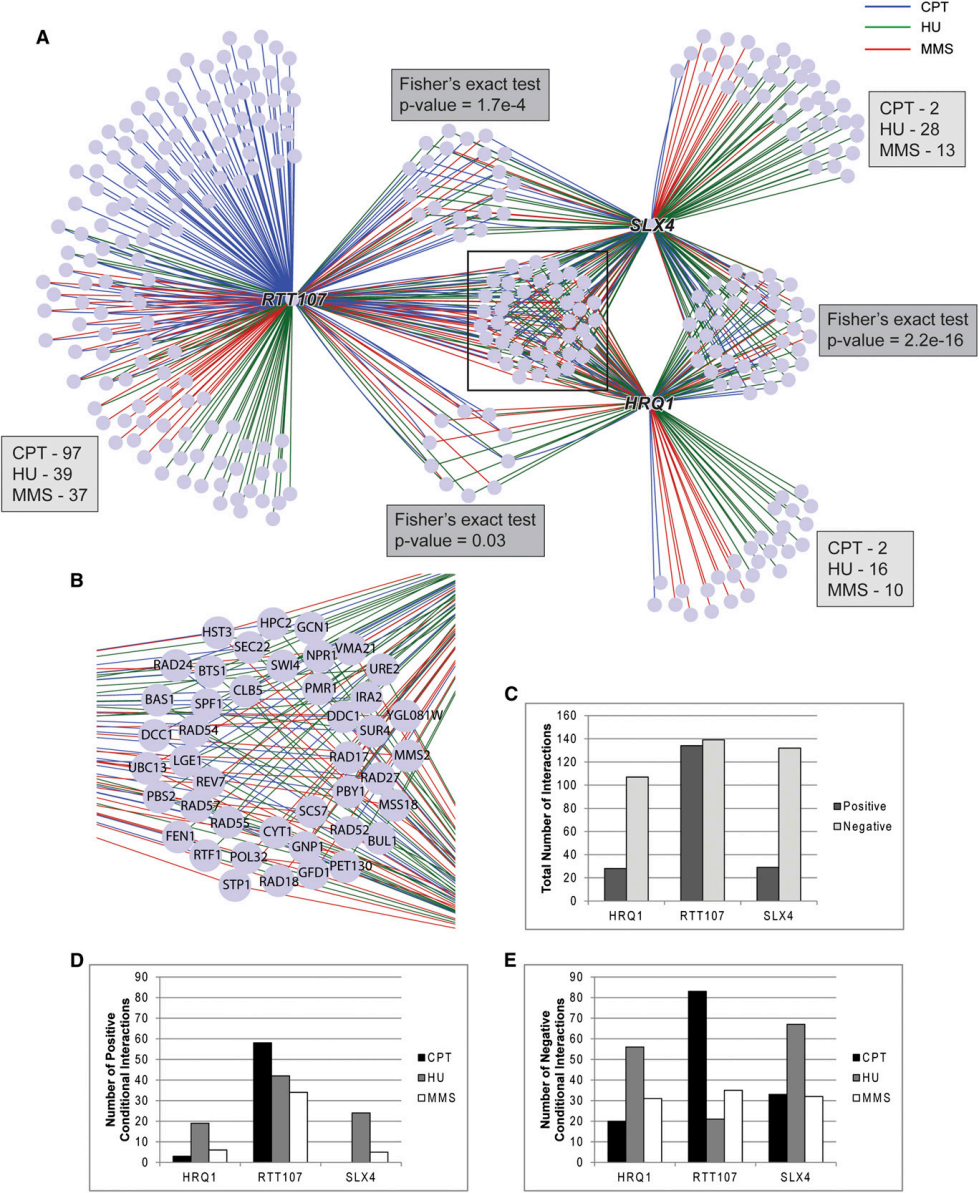
*RTT107* had more significant genetic interactions than *SLX4* or *HRQ1*. (A) Visualization of all the significant genetic interactions. Nodes represent query or array genes, and edges are colored by the drug condition that the interaction occurred in. Blue represents CPT, green represents HU, and red represents MMS. The numbers of unique interactions for each query gene are labeled for each drug condition. Indicated *p*-values are from Fisher’s exact tests (greater) of the genes that interact only with two out of the three query genes. (B) Enlarged view of a subset of the network indicated by the black box in (A). (C) *RTT107* had more positive genetic interactions than *SLX4* or *HRQ1*. *RTT107* had more (D) positive and (E) negative genetic interactions in CPT than *SLX4* or *HRQ1*. CPT, camptothecin; HU, hydroxyurea; MMS, methyl methane-sulfonate.

We visualized the conditional genetic interactions in a network in which nodes represented query or array genes and edges represented significant conditional genetic interactions. The edges were colored according to the drug condition that the genetic interaction occurred in (Figure 4A). As revealed by the network map, there was a subset of genes that interacted with all three query genes, suggesting that they play a more general role in the DNA damage response. These included the homologous recombination genes *RAD52*, *RAD55*, and *RAD57* (Figure 4B). Aside from this group of genes, there were also subsets that interacted with only two out of the three query genes. *HRQ1* and *SLX4* shared the greatest number of interacting genes, and this represented a significant overlap between these two groups (Fisher’s exact test, greater, *p*-value = 2.2 × 10^−16^, Figure 4A). Further supporting shared functions of *RTT107* and *SLX4*, there was also a significant overlap between their interacting genes (Fisher’s exact test, greater, *p*-value = 1.7 × 10^−4^). Conversely, each query gene had unique genetic interactions, and *RTT107* had the greatest number of these (Figure 4A). Whereas the majority of the unique genetic interactions with *RTT107* occurred under CPT conditions, *HRQ1* and *SLX4* had minimal numbers of unique interactions in CPT.

**Figure 5:**
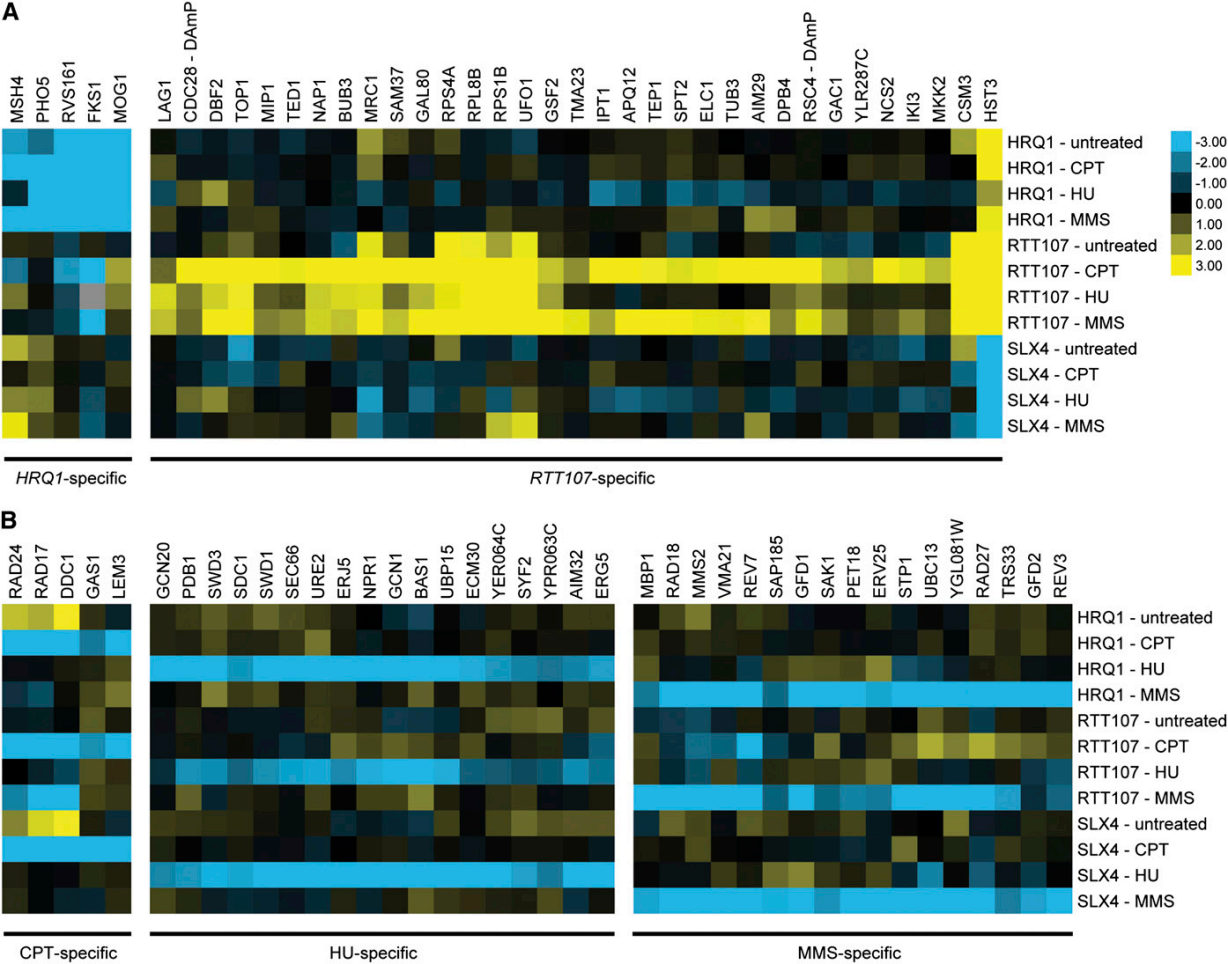
Different patterns of genetic interactions were observed for genes that significantly changed their interactions in response to DNA-damaging conditions. Shown are subsets of conditional epistatic miniarray profiling (cE-MAP) data. Blue and yellow represent negative and positive genetic interactions, respectively. (A) Some genetic interactions were specific for the query genes. (B) Other genetic interactions were specific to the drug condition and were common across all query genes.

When comparing the total number of genetic interactions, we found that *RTT107* had many more positively interacting genes than either *SLX4* or *HRQ1* (Figure 4C). After these interactions were separated into each drug condition, it became clear that the biggest difference in interactions occurred during exposure to CPT for both positive and negative interactions (Figure 4, D and E). Taken together, these data suggest that Rtt107 plays an important role in responding to protein-bound nicks induced by CPT. To support this, *RTT107* also had a strong positive/epistatic genetic interaction with *TOP1*, the molecular target of CPT, whereas this interaction was absent for *SLX4* and *HRQ1* (Figure 5A).

### Genes with condition-specific interactions were enriched for functions in the DNA damage response

To further analyze the functions of *RTT107*, *SLX4*, and *HRQ1* revealed by the conditional genetic interactions, we looked at the enrichment of GO terms using the Database for Annotation, Visualization, and Integrated Discovery (Dennis *et al.* 2003). For each query gene, all the genes that had significant conditional interactions in all three DNA-damaging conditions were analyzed for GO term enrichment. Because the library of genes tested with our cE-MAP approach was already enriched for nuclear function, we used this list of genes as the background for the analysis rather than the whole set of genes in the yeast genome. All the significantly enriched GO terms were related to the DNA damage response as expected (Table 2), indicating that the treatments induced DNA damage specifically and not a general stress response. However, the specific enriched processes differed between the three query genes. Both *SLX4* and *HRQ1* were enriched for processes related to DNA metabolism and repair, and postreplication repair was one of the most overrepresented terms (5.45- and 6.42-fold enrichment, respectively). Notably, the lists of enriched GO terms for *SLX4* and *HRQ1* were almost identical, further suggesting that these two genes have similar functions in the DNA damage response. In contrast, *RTT107* was significantly enriched only for one GO term, cell cycle checkpoint (2.95 fold enrichment, *q* < 0.03), although many of the GO terms that did not meet the significance cutoff were also related to the cell cycle (data not shown).

**Table 2:** GO terms that were significantly enriched in the list of genetically interacting genes under all conditions using DAVID

Query	GO Term	Fold Enrichment	*q*-value (Benjamini)
*RTT107*	Cell-cycle checkpoint	2.95	2.56E-02
*SLX4*	DNA metabolic process	2.54	6.22E-07
	Response to DNA damage stimulus	2.53	1.87E-05
	DNA recombination	4.09	1.29E-05
	DNA repair	2.59	4.70E-05
	Recombinational repair	4.91	6.39E-04
	DNA replication	3.03	7.59E-04
	Postreplication repair	5.45	1.94E-02
	Cellular response to stress	1.78	2.31E-02
*HRQ1*	DNA metabolic process	2.83	8.29E-08
	DNA recombination	4.64	7.05E-06
	Response to DNA damage stimulus	2.77	5.32E-06
	DNA repair	2.90	8.83E-06
	DNA replication	3.44	2.28E-04
	Cellular response to stress	2.08	9.93E-04
	Postreplication repair	6.52	5.21E-03
	Recombinational repair	4.89	5.12E-03
	DNA-dependent DNA replication	3.91	6.35E-03
	Double-strand break repair	3.67	1.13E-02
	Double-strand break repair via single-strand annealing	7.34	2.97E-02

GO, Gene Ontology; DAVID, Database for Annotation, Visualization, and Integrated Discovery.

### Deletion of *HST3* and *MRC1* suppressed the DNA damage sensitivity of *rtt107*∆ but not *slx4*∆ mutants

Hierarchical clustering of the significantly interacting genes revealed several patterns of genetic interaction profiles. There were sets of genes that were specific for the query gene, regardless of the DNA-damaging agent (Figure 5A). Intriguingly, there were certain genes that showed strong positive interactions with *RTT107* but negative interactions with *SLX4*, such as *HST3* and *MRC1*, further supporting the idea that Rtt107 and Slx4 have unique functions.

Conversely, other sets of genes were specific for the DNA-damaging agent and interacted with all three query genes under that condition (Figure 5B). For example, the CPT-specific genes included *RAD24*, *RAD17*, and *DDC1*, which encode for components of the 9-1-1 checkpoint clamp and RFC loader complex (Majka and Burgers. 2007). The MMS-specific genes included multiple components of the translesion synthesis pathway, such as *REV3*, *REV7*, and *RAD18* (Sharma *et al.* 2013). Unexpectedly, the HU-specific genes included several transcription-related genes such as *SWD1*, *SWD3*, and *SDC1*.

**Figure 6:**
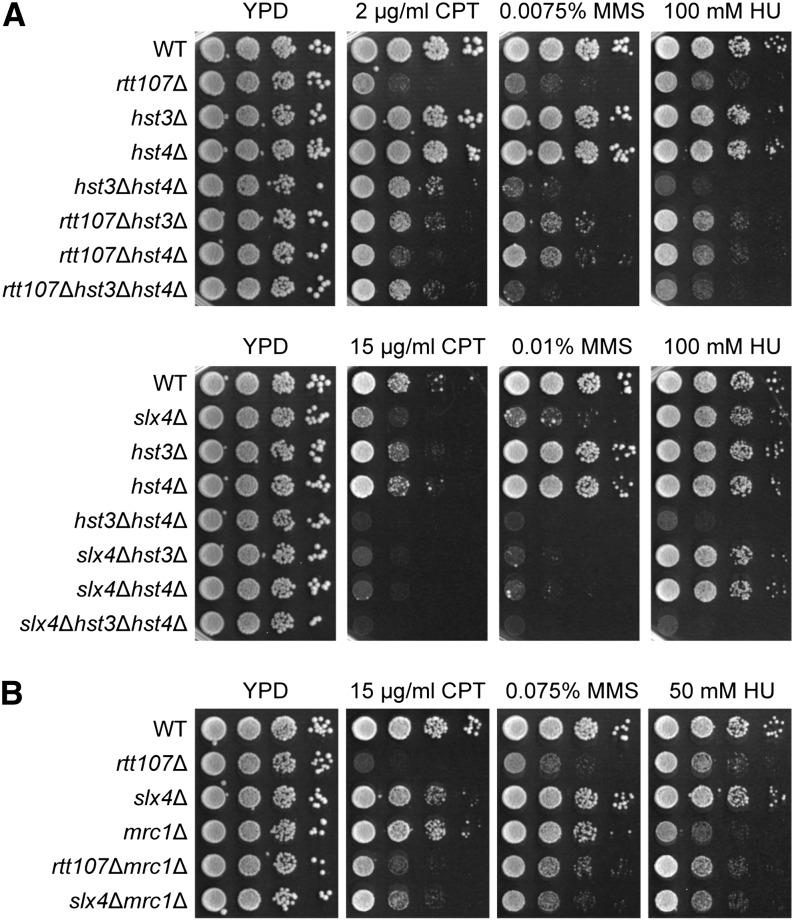
Positive S-scores from the cE-MAP data were based on suppression of DNA damage sensitivity of *rtt107*∆ mutants. 10-fold serial dilutions of the indicated strains were plated onto media containing the indicated drugs. (A) Deletion of *HST3* and *HST4* suppressed the DNA damage sensitivity of *rtt107*∆ mutants but aggravated the sensitivity of *slx4*∆ mutants. (B) Similarly, deletion of *MRC1* suppressed the DNA damage sensitivity of *rtt107*∆ mutants but aggravated the sensitivity of *slx4*∆ mutants.

Positive S-scores indicate that the double mutant exhibits better fitness than expected (multiplicative product of single mutants’ fitness), but it does not differentiate between suppression and epistasis. To further investigate the nature of the genetic interaction between *HST3* and *RTT107* and *SLX4*, we independently constructed deletion mutants and extended the analysis to include *HST4*, which was not on the E-MAP library. Hst3 and Hst4 are protein deacetylases that are both responsible for removing histone H3 K56 acetylation, thereby affecting replicative lifespan and response to DNA damage (Miller *et al.* 2006). Deletion of *HST3* clearly suppressed the DNA damage sensitivity of *rtt107*Δ mutants in all three drugs tested, albeit to a lesser extent in HU (Figure 6A). Confirming the striking opposite interactions of *RTT107* and *SLX4* observed in the E-MAP, *HST3* and *SLX4* showed a synergistic interaction in CPT and MMS, but not HU. In general, *HST4* showed the same genetic interaction profile as *HST3*. However, the *hst3*∆*hst4*∆*rtt107*∆ triple mutant showed a variable phenotype depending on the DNA-damaging agent, and this differed from the *hst3*∆*rtt107*∆ or *hst4*∆*rtt107*∆ double mutants, portraying a complex relationship between *HST3* and *HST4* in this genetic interaction.

As a second example of characterizing positive interactions, we focused on *MRC1*, a gene encoding for an S-phase checkpoint adaptor (Tanaka 2010), which also showed this pattern of opposite genetic interactions with *RTT107* and *SLX4*. Using independently constructed deletion mutants, we observed that deletion of *MRC1* suppressed the DNA damage sensitivity of *rtt107*Δ mutants to CPT and MMS (Figure 6B). Interestingly, the converse was observed in the case of HU, in that deletion of *RTT107* mildly suppressed the sensitivity of *mrc1*∆ mutants. In contrast, the *slx4*∆*mrc1*∆ double mutant was clearly more sensitive to CPT and MMS than the *slx4*∆ single mutant, although the deletion of *SLX4* mildly suppressed the HU sensitivity of the *mrc1*∆ single mutant.

## Discussion

In this study we used cE-MAP to generate conditional genetic interaction profiles for *RTT107*, *SLX4*, and *HRQ1*, to further investigate their functions in the DNA damage response. We tested CPT, HU, and MMS, which elicited specific genetic interactions with each of the query genes, and the network of interactions observed provides insight into the mechanisms of both the DNA-damaging agent and the query genes. Furthermore, we validated two specific examples of genetic interactions emerging from the cE-MAP in direct genetic tests and identified a novel genetic suppression in each case.

A critical component of the cE-MAP approach was the use of DNA-damaging agents. Because we are interested in interrogating the DNA damage response functions of the query genes, it was crucial to evaluate the genetic interactions in conditions when those functions are active. Testing three different DNA-damaging agents also provided an opportunity to compare between the responses of the genetic interaction network to each type of DNA insult.

We observed sets of genes that showed significant interactions under each specific drug condition. For each drug condition there was different DNA damage response pathways represented within the sets of genes, supporting the idea that the cell responds specifically to different types of DNA lesions. The CPT-specific genes included components of the 9-1-1 checkpoint clamp and RFC loader complex, suggesting that the DNA damage response to protein adducts involves this component of the checkpoint response (Majka and Burgers. 2007). The MMS-specific genes included multiple components of the translesion synthesis pathway, which is one of the pathways in postreplication repair that allows cells to replicate past damaged bases or bulky adducts (Sharma *et al.* 2013). These data suggest that it also plays a role in bypassing alkylated bases. The HU-specific genes included several transcription-related genes, which was unexpected, but could be explained by an indirect effect of the mechanism of HU, which depletes the deoxynucleotide triphosphate pool. Interestingly, one of the HU-specific genes, *YER064C*, is relatively uncharacterized but was recently shown to change its cellular localization upon exposure to HU, suggesting a role for this gene in response to replication stress (Tkach *et al.* 2012). Consistent with our study, the previously published E-MAP analysis of the DNA damage response revealed that genes showing significant interactions in CPT are enriched for function in the DNA damage checkpoint, whereas significant genes in MMS are enriched for post-replication repair (Guenole *et al.* 2013).

In contrast to the drug-specific genes, there were also sets of genes that showed unique interactions with each specific query gene. These interactions suggested that the query genes we interrogated have distinct functions in the DNA damage response. It is of particular interest that *RTT107* and *SLX4* shared only a subset of genetic interactions, given that the Rtt107 and Slx4 proteins exist as a complex in the cell (Roberts *et al.* 2006). An attractive model is that there are different pools of Rtt107 and Slx4 protein complexes that contribute to specific functions, since Rtt107’s interaction with SMC5/6 and Slx4’s interaction with Slx1 are independent of each other (Roberts *et al.* 2006; Leung *et al.* 2011). The human homologs of Rtt107 and Slx4, PTIP and SLX4, respectively, also have many distinct functions. While PTIP is involved in the DNA damage signaling cascades and DNA repair pathway choice (Gong *et al.* 2009; Wu *et al.* 2009; Callen *et al.* 2013), SLX4 has roles in Holliday junction resolution and telomere length regulation (Castor *et al.* 2013; Garner *et al.* 2013; Wan *et al.* 2013; Wilson *et al.* 2013; Wyatt *et al.* 2013). The data from this cE-MAP provide an opportunity to further elucidate the unique functions of Rtt107 and Slx4, which may be further dissected into responses to different DNA lesions.

The cE-MAP data also provided more insight into the function of the helicase Hrq1, which has only been preliminarily characterized (Kwon *et al.* 2012; Choi *et al.* 2013; Bochman *et al.* 2014). Interestingly, the genetic interaction profile of *HRQ1* correlated more closely to *SLX4* than *RTT107*, and the sets of genes that interacted with *SLX4* or *HRQ1* overlapped significantly. In addition, GO analysis of the significantly interacting genes returned almost identical GO terms for *HRQ1* and *SLX4*. The close relationship between *HRQ1* and *SLX4* revealed by the cE-MAP data are supported by previous studies showing that *SLX4* is synthetic lethal with *SGS1*, the major RecQ helicase in *S. cerevisiae* (Mullen *et al.* 2001). Moreover, there is some evidence suggesting that Hrq1 and Slx4 are both involved in interstrand crosslink repair and suppression of telomere addition (Zhang *et al.* 2006; Ward *et al.* 2012; Bochman *et al.* 2014). However, *slx4*∆ and *hrq1*∆ mutants display different sensitivities to DNA-damaging agents, indicating they function separately as well (Flott and Rouse. 2005; Choi *et al.* 2013; Bochman *et al.* 2014). Further experiments are needed to determine the roles of Hrq1 and Slx4 in DNA structure maintenance and the nature of their relationship in these functions. Possible routes of inquiry can be suggested by additional examination of the genetic data.

We followed up on two genes that had particularly striking conditional genetic interactions. Both *HST3* and *MRC1* showed a strong positive genetic interaction with *RTT107* but a negative interaction with *SLX4*. Although Rtt107 and Slx4 form a complex, these genetic interactions suggest that not only do Rtt107 and Slx4 have independent functions, but they may have opposing functions in these contexts. Using a direct genetic test, we found that deletion of *HST3*, as well as *HST4*, suppressed the DNA damage sensitivity of *rtt107*∆ mutants. The known target of the Hst3 and Hst4 deacetylases is H3 K56 acetylation (H3 K56ac). Whereas deletion of *HST3* alone causes an increase in H3 K56ac, deletion of *HST4* alone does not change the acetylation levels, and only in the double mutant are all H3 molecules completely acetylated (Celic *et al.* 2006). Intriguingly, the suppression of the *rtt107*∆ mutant phenotype in CPT was observed upon deletion of *HST3* or both *HST3* and *HST4*, but not *HST4* alone. In contrast, deletion of either *HST3* or *HST4* alone was sufficient to suppress the DNA damage sensitivity of *rtt107*∆ mutants to MMS and HU. Based on this data, we speculate that the deacetylation of H3 K56ac may be important to the genetic interaction in CPT, whereas it is not relevant in MMS or HU conditions, rather there may be a different function or target of Hst3 and Hst4 involved. Similarly for the genetic interaction with *SLX4*, deletion of *HST3* or *HST4* alone exhibited the same phenotype, thus suggesting that deacetylation of H3 K56ac is not involved.

We also validated the genetic interaction with *MRC1* and found that deletion of *MRC1* suppressed the DNA damage sensitivity of *rtt107*∆ mutants in CPT and MMS but aggravated the sensitivity of *slx4*∆ mutants. Interestingly, the situation was different in HU, where deletion of either *RTT107* or *SLX4* mildly suppressed the sensitivity of *mrc1*∆ mutants. This finding is consistent with a model proposed by a previous study suggesting that Rtt107 and Slx4 inhibit the checkpoint adaptor protein Rad9, which is normally not important in replication stress, but becomes crucial in the absence of Mrc1 (Ohouo *et al.* 2013). However, this model does not explain the genetic interactions observed in CPT and MMS and reflects the distinct responses to various types of DNA lesions, as well as the multiple functions of DNA damage response proteins.

Our study contributes to the growing of body of data that has mapped genetic interactions in response to DNA damage and further validates it as a fruitful approach that reveals condition-specific functions and pathways in the cell. There remains much ground to be covered as we have only started to characterize the pathways specific for the multitude of environmental conditions that affect all living organisms.

## Supplementary Material

Supporting Information
