# Supplementary material for: Conditional Genetic Interactions of RTT107, SLX4, and HRQ1 Reveal Dynamic Networks upon DNA Damage in S. cerevisiae
Source: G3 (Bethesda). 2014 Apr 2;4(6):1059–69. doi: 10.1534/g3.114.011205 (PMC4065249; doi:10.1534/g3.114.011205)
Supplement: Supporting Information [file supp_4_6_1059__index.html]

Conditional Genetic Interactions of RTT107, SLX4, and HRQ1 Reveal Dynamic Networks upon DNA Damage in S. cerevisiae — Supporting Information 

# Conditional Genetic Interactions of *RTT107*, *SLX4*, and *HRQ1* Reveal Dynamic Networks upon DNA Damage in *S. cerevisiae*

## Supporting Information for Leung *et al.*, 2014

**Files in this Data Supplement:**

- Supporting Information - Figure S1, Table S1, and File S1 (PDF, 311 KB)
- Figure S1 - Distribution of S-scores differed between conditions or between query genes, as shown by cumulative probability plots. (PDF, 649 KB)
- Table S1 - Yeast strains used in this study. (PDF, 146 KB)
- File S1 - Complete cE-MAP profiles. (Includes the original profiles with both drug concentrations and the averaged profiles.) (.xlsx, 428 KB)
