# Supplementary material for: Conditional Genetic Interactions of RTT107, SLX4, and HRQ1 Reveal Dynamic Networks upon DNA Damage in S. cerevisiae
Source: G3 (Bethesda). 2014 Apr 2;4(6):1059–69. doi: 10.1534/g3.114.011205 (PMC4065249; doi:10.1534/g3.114.011205)
Supplement: Supporting Information [file supp_g3.114.011205_011205SI.pdf]

G3: Genes | Genomes | Genetics

Conditional Genetic Interactions of *RTT107*, *SLX4*, and *HRQ1* Reveal Dynamic Networks Upon DNA Damage in *S. Cerevisiae*

Grace P. Leung[*,1], Maria J. Aristizabal[*,1], Nevan J. Krogan[†], Michael S. Kobor[*2]

[1]These authors contributed equally to this work.

[*]Centre for Molecular Medicine and Therapeutics, Child and Family Research Institute,
Department of Medical Genetics, University of British Columbia, Vancouver, B.C. Canada V5Z 4H4
[†]Department of Cellular and Molecular Pharmacology, California Institute for Quantitative Biomedical Research, University of California, San Francisco, San Francisco, California 94158

[2]Corresponding author: Centre for Molecular Medicine and Therapeutics, 950 West 28th Avenue, Vancouver, B.C., Canada, V5Z 4H4. Email: msk@cmmt.ubc.ca

DOI: 10.1534/g3.114.011205

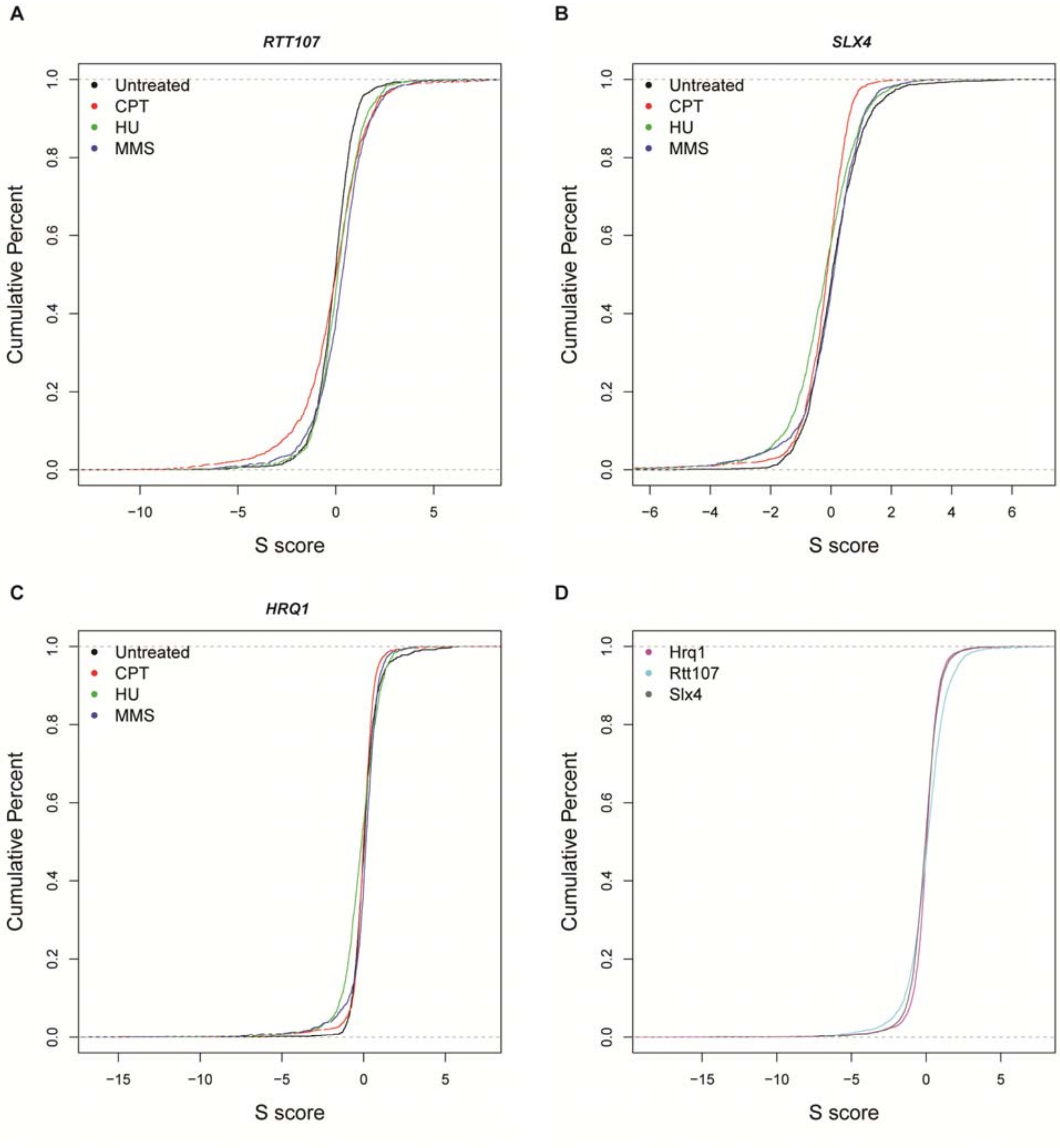

Figure S1. Distribution of S-scores differed between conditions or between query genes, as shown by cumulative probability plots. (A) S-scores for *RTT107*. Kolmogorov-Smirnov test, two-sided, p-values for untreated vs CPT = 6.4e-09, untreated vs HU = 1.4e-07, untreated vs MMS < 2.2e-16. (B) S-scores for *SLX4*. Kolmogorov-Smirnov test, two-sided, p-values for untreated vs CPT = 5.6e-16, untreated vs HU = 4.9e-10, untreated vs MMS = 0.14. (C) S-scores for *HRQ1*. Kolmogorov-Smirnov test, two-sided, p-values for untreated vs CPT = 2.5e-03, untreated vs HU < 2.2e-16, untreated vs MMS = 7.0e-05. (D) Combined S-scores for all conditions for each query gene. Kolmogorov-Smirnov test, two-sided, p-values for *RTT107* vs *HRQ1* < 2.2e-16, *RTT107* vs *SLX4* < 2.2e-16, *HRQ1* vs *SLX4* = 7.8e-16.

Table S1 Yeast strains used in this study.

Strain	Relevant Genotype	Background
MKY1649	*Matα his3Δ1 leu2Δ0 LYS2+ met15Δ0 ura3Δ0 Δcan1::MATaPr-HIS3 Δlyp1::MATαPr-LEU2 rtt107::NATMX6*	BY4742
MKY1650	*Matα his3Δ1 leu2Δ0 LYS2+ met15Δ0 ura3Δ0 Δcan1::MATaPr-HIS3 Δlyp1::MATαPr-LEU2 slx4::NATMX6*	BY4742
MKY1651	*Matα his3Δ1 leu2Δ0 LYS2+ met15Δ0 ura3Δ0 Δcan1::MATaPr-HIS3 Δlyp1::MATαPr-LEU2 hrq1::NATMX6*	BY4742
MKY5	*MATα ade2-1 can1-100 his3-11 leu2-3,112 trp1-1 ura3-1*	W303-1A
MKY7	*MAT**a** ade2-1 can1-100 his3-11 leu2-3,112 trp1-1 ura3-1*	W303-1A
MKY1652	MKY5, *rtt107::KANMX6*	W303-1A
MKY1653	MKY5, *dot1::HIS3*	W303-1A
MKY959	MKY5, *rtt107::KANMX6 dot1::HIS3*	W303-1A
MKY1654	MKY5, *bre1::HYGMX*	W303-1A
MKY1655	MKY5, *rtt107::KANMX6 bre1::HYGMX*	W303-1A
MKY1656	MKY5, *slx4::HYGMX*	W303-1A
MKY1657	MKY5, *hrq1::NATMX6*	W303-1A
MKY1658	MKY7, *slx4::NATMX6*	W303-1A
MKY1659	MKY5, *mrc1::HIS3*	W303-1A
MKY1660	MKY5, *mrc1::HIS3 rtt107::KANMX6*	W303-1A
MKY1661	MKY5, *mrc1::HIS3 slx4::KANMX6*	W303-1A
MKY1662	MKY5, *hst3::HIS3*	W303-1A
MKY1663	MKY5, *hst4::HYGMX*	W303-1A
MKY1664	MKY5, *hst3::HIS3 slx4::KANMX6*	W303-1A
MKY1665	MKY5, *hst3::HIS3 rtt107::KANMX6*	W303-1A
MKY1666	MKY5, *hst4::HYGMX rtt107::KANMX6*	W303-1A
MKY1667	MKY5, *hst4::HYGMX slx4::KANMX6*	W303-1A
MKY1668	MKY5, *hst3::HIS3 hst4::HYGMX*	W303-1A
MKY1669	MKY5, *hst3::HIS3 hst4::HYGMX rtt107::KANMX6*	W303-1A
MKY1670	MKY5, *lys2Δ hst3::HIS3 hst4::HYGMX slx4::KANMX6*	W303-1A

File S1

Complete cE-MAP profiles. (Includes the original profiles with both drug concentrations and the averaged profiles.)

File S1 is available for download as an Excel file at http://www.g3journal.org/lookup/suppl/doi:10.1534/g3.114.011205/-/DC1
