# Supplementary material for: Conditional Genetic Interactions of RTT107, SLX4, and HRQ1 Reveal Dynamic Networks upon DNA Damage in S. cerevisiae
Source: G3 (Bethesda). 2014 Apr 2;4(6):1059–69. doi: 10.1534/g3.114.011205 (PMC4065249; doi:10.1534/g3.114.011205)
Supplement: Supporting Information [file supp_g3.114.011205_FigureS1.pdf]

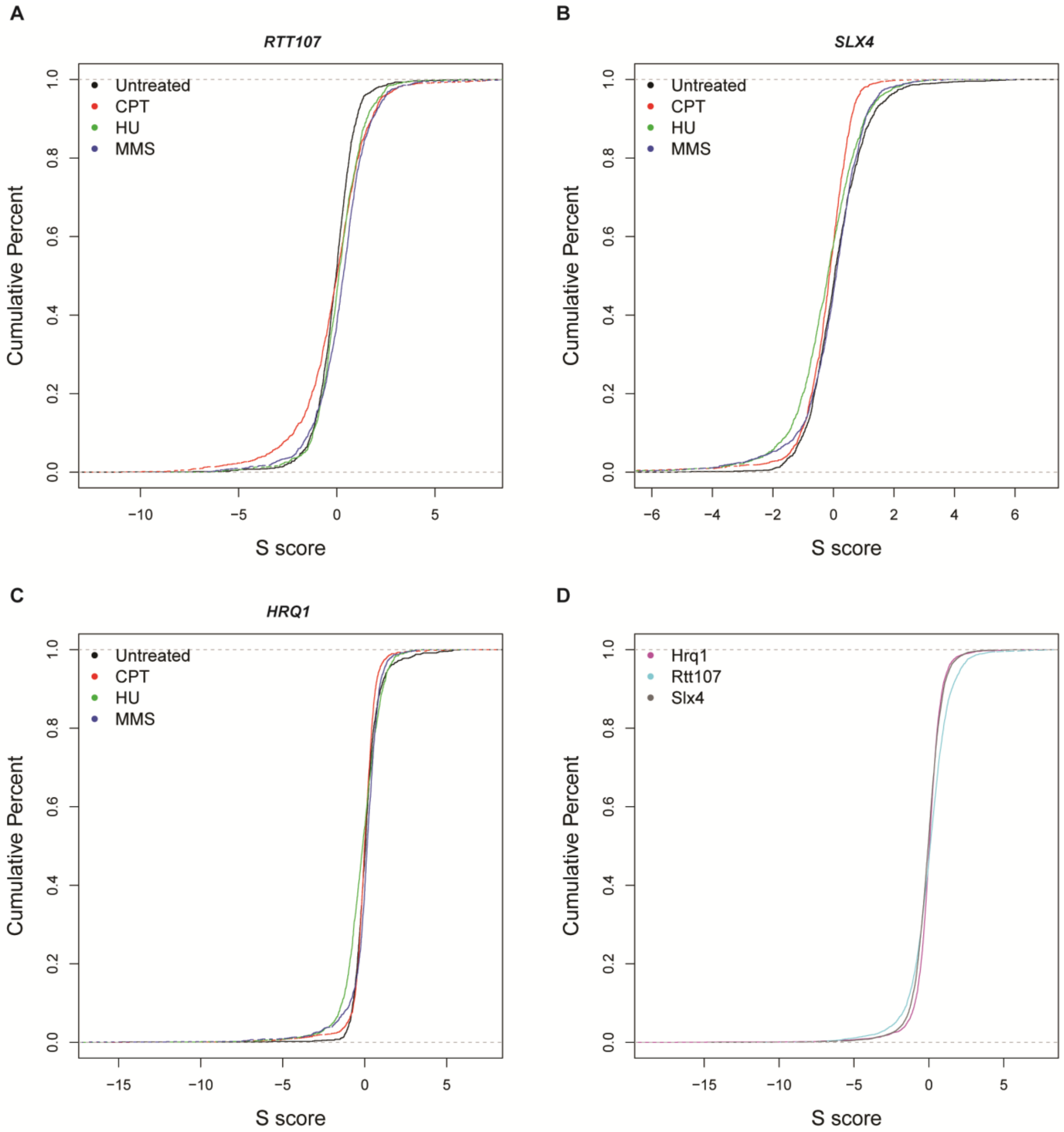

Figure S1. Distribution of S-scores differed between conditions or between query genes, as shown by cumulative probability plots. (A) S-scores for *RTT107*. Kolmogorov-Smirnov test, two-sided, p-values for untreated vs CPT = 6.4e-09, untreated vs HU = 1.4e-07, untreated vs MMS < 2.2e-16. (B) S-scores for *SLX4*. Kolmogorov-Smirnov test, two-sided, p-values for untreated vs CPT = 5.6e-16, untreated vs HU = 4.9e-10, untreated vs MMS = 0.14. (C) S-scores for *HRQ1*. Kolmogorov-Smirnov test, two-sided, p-values for untreated vs CPT = 2.5e-03, untreated vs HU < 2.2e-16, untreated vs MMS = 7.0e-05. (D) Combined S-scores for all conditions for each query gene. Kolmogorov-Smirnov test, two-sided, p-values for *RTT107* vs *HRQ1* < 2.2e-16, *RTT107* vs *SLX4* < 2.2e-16, *HRQ1* vs *SLX4* = 7.8e-16.
