# Supplementary material for: Conditional Genetic Interactions of RTT107, SLX4, and HRQ1 Reveal Dynamic Networks upon DNA Damage in S. cerevisiae
Source: G3 (Bethesda). 2014 Apr 2;4(6):1059–69. doi: 10.1534/g3.114.011205 (PMC4065249; doi:10.1534/g3.114.011205)
Supplement: Supporting Information [file supp_g3.114.011205_TableS1.pdf]

Table S1 Yeast strains used in this study.

Strain	Relevant Genotype	Background
MKY1649	*Matα his3Δ1 leu2Δ0 LYS2+ met15Δ0 ura3Δ0 Δcan1::MATaPr-HIS3 Δlyp1::MATαPr-LEU2 rtt107::NATMX6*	BY4742
MKY1650	*Matα his3Δ1 leu2Δ0 LYS2+ met15Δ0 ura3Δ0 Δcan1::MATaPr-HIS3 Δlyp1::MATαPr-LEU2 slx4::NATMX6*	BY4742
MKY1651	*Matα his3Δ1 leu2Δ0 LYS2+ met15Δ0 ura3Δ0 Δcan1::MATaPr-HIS3 Δlyp1::MATαPr-LEU2 hrq1::NATMX6*	BY4742
MKY5	*MATα ade2-1 can1-100 his3-11 leu2-3,112 trp1-1 ura3-1*	W303-1A
MKY7	*MAT**a** ade2-1 can1-100 his3-11 leu2-3,112 trp1-1 ura3-1*	W303-1A
MKY1652	MKY5, *rtt107::KANMX6*	W303-1A
MKY1653	MKY5, *dot1::HIS3*	W303-1A
MKY959	MKY5, *rtt107::KANMX6 dot1::HIS3*	W303-1A
MKY1654	MKY5, *bre1::HYGMX*	W303-1A
MKY1655	MKY5, *rtt107::KANMX6 bre1::HYGMX*	W303-1A
MKY1656	MKY5, *slx4::HYGMX*	W303-1A
MKY1657	MKY5, *hrq1::NATMX6*	W303-1A
MKY1658	MKY7, *slx4::NATMX6*	W303-1A
MKY1659	MKY5, *mrc1::HIS3*	W303-1A
MKY1660	MKY5, *mrc1::HIS3 rtt107::KANMX6*	W303-1A
MKY1661	MKY5, *mrc1::HIS3 slx4::KANMX6*	W303-1A
MKY1662	MKY5, *hst3::HIS3*	W303-1A
MKY1663	MKY5, *hst4::HYGMX*	W303-1A
MKY1664	MKY5, *hst3::HIS3 slx4::KANMX6*	W303-1A
MKY1665	MKY5, *hst3::HIS3 rtt107::KANMX6*	W303-1A
MKY1666	MKY5, *hst4::HYGMX rtt107::KANMX6*	W303-1A
MKY1667	MKY5, *hst4::HYGMX slx4::KANMX6*	W303-1A
MKY1668	MKY5, *hst3::HIS3 hst4::HYGMX*	W303-1A
MKY1669	MKY5, *hst3::HIS3 hst4::HYGMX rtt107::KANMX6*	W303-1A
MKY1670	MKY5, *lys2Δ hst3::HIS3 hst4::HYGMX slx4::KANMX6*	W303-1A
